# High-precision radiotherapy of motor deficits due to metastatic spinal cord compression (PRE-MODE): a multicenter phase 2 study

**DOI:** 10.1186/s12885-017-3844-x

**Published:** 2017-12-04

**Authors:** Dirk Rades, Jon Cacicedo, Antonio J. Conde-Moreno, Claudia Doemer, Jürgen Dunst, Darejan Lomidze, Barbara Segedin, Denise Olbrich, Niels Henrik Holländer

**Affiliations:** 1 Department of Radiation Oncology, University of Lübeck, Ratzeburger Allee 160, D-23562 Lübeck, Germany; 2 Department of Radiation Oncology, Cruces University Hospital, Barakaldo, Vizcaya Spain; 3 Department of Radiation Oncology, Consorcio Hospital Provincial de Castellón, Castellón, Spain; 4 Department of Radiation Oncology, Christian-Albrechts University Kiel, Kiel, Germany; 5 Radiation Oncology Department, High Technology Medical Center, University Clinic Tbilisi, Tbilisi, Georgia; 6 Department of Radiotherapy, Institute of Oncology Ljubljana, Ljubljana, Slovenia; 7 Centre for Clinical Trials Lübeck, Lübeck, Germany; 8 Department of Oncology, Zealand University Hospital, Naestved, Denmark

**Keywords:** Metastatic spinal cord compression, Volumetric modulated arc therapy, Stereotactic body radiotherapy, Local progression-free survival, Motor function, Overall survival, Pain, Quality of life

## Abstract

**Background:**

For metastatic spinal cord compression (MSCC), conventional radiotherapy with 10 × 3 Gy in 2 weeks results in better local progression-free survival (LPFS) than 5 × 4 Gy in 1 week. Since patients with MSCC are often significantly impaired, an overall treatment time of 1 week would be preferable if resulting in similar outcomes as longer programs. This may be achieved with 5 × 5 Gy in 1 week, since the biologically effective dose is similar to 10 × 3 Gy. It can be expected that 5 × 5 Gy (like 10 × 3) Gy results in better LPFS than 5 × 4 Gy in 1 week.

**Methods/Design:**

This phase 2 study investigates LPFS after high-precision RT with 5 × 5 Gy in 1 week. LPFS is defined as freedom from both progression of motor deficits during RT and new or progressive motor deficits dur to an in-field recurrence of MSCC following RT. Considering the tolerance dose of the spinal cord, 5 × 5 Gy can be safely administered with high-precision radiotherapy such as volumetric modulated arc therapy (VMAT) or stereotactic body radiotherapy (SBRT). Maximum dose to the spinal cord should not exceed 101.5% of the prescribed dose to keep the risk of radiation myelopathy below 0.03%. Primary endpoint is LPFS at 6 months following radiotherapy; secondary endpoints include motor function/ability to walk, sensory function, sphincter dysfunction, LPFS directly and 1 and 3 months following radiotherapy, overall survival, pain relief, quality of life and toxicity. Follow-up visits will be performed directly and at 1, 3 and 6 months following radiotherapy. After completion of this phase 2 study, patients will be compared to a historical control group receiving conventional radiotherapy with 5 × 4 Gy in 1 week. Forty-four patients will be included assuming 5 × 5 Gy will provide the same benefit in LPFS when compared to 5 × 4 Gy as reported for 10 × 3 Gy.

**Discussion:**

If superiority regarding LPFS is shown for high-precision radiotherapy with 5 × 5 Gy when compared to conventional radiotherapy with 5 × 4 Gy, patients with MSCC would benefit from 5 × 5 Gy, since high LPFS rates could be achieved with 1 week of radiotherapy instead of 2 weeks (10 × 3 Gy).

**Trial registration:**

clinicaltrials.gov NCT03070431. Registered 27 February 2017.

## Background

Metastatic spinal cord compression (MSCC) occurs in 5-10% of all cancer patients during the course of their disease [1, 2]. Radiotherapy (RT) alone is the most common treatment used for the treatment of MSCC worldwide. However, the most appropriate radiation schedule is still a matter of debate. The survival prognosis of many patients with MSCC is poor [1–3]. Every RT session may be associated with discomfort for the often significantly impaired patients in a palliative situation, in particular regarding the transport to the radiation oncology department and the patient’s positioning on the treatment couch. Thus, a more patient convenient radiation schedule with a short overall treatment time (short-course radiotherapy such as 5 × 4 Gy in 1 week) would be preferable if it was as effective as the most commonly used radiation schedule for MSCC, 10 × 3 Gy in 2 weeks. Previous studies have shown that 5 × 4 Gy in 1 week and 10 × 3 Gy in 2 weeks are similarly effective with respect to improvement of motor function [3, 4].

However, a prospective non-randomized study has demonstrated that longer-course radiotherapy programs such as 10 × 3 Gy in 2 weeks resulted in better local progression-free survival (LPFS) than short-course programs such as 5 × 4 Gy in 1 week [5]. LPFS was defined as freedom from both progression of motor deficits during RT and from an in-field recurrence of MSCC following RT (in-field recurrence = motor deficits due to a recurrence of MSCC in the previously irradiated parts of the spine). The LPFS rates at 6 months were 86% after longer-course RT and 67% after short-course RT, respectively (*p* = 0.034). Local progression of MSCC is a serious situation, since spinal surgery or a second course of radiotherapy in the same area of the spinal cord may not be possible. Therefore, such a progression must be avoided.

The ideal RT schedule for MSCC would be both short and effective in improving LPFS. The biological effect of radiotherapy depends on both the total dose and the dose per fraction [6]. The biologically effective doses of different RT schedules can be compared by calculating the equivalent dose in 2 Gy fractions (EQD2) [7]. The EQD2 with respect to tumor cell kill (alpha/beta value of 10 Gy) is 23.3 Gy for 5 × 4 Gy and 32.5 Gy for 10 × 3 Gy, respectively. RT of MSCC can be intensified with the use of high-precision techniques such as volumetric modulated arc therapy (VMAT) and stereotactic body radiotherapy (SBRT) without compromising the tolerance doses of the spinal cord and the vertebral bone [8–11]. Since the EDQ2 of high-precision RT with 5 × 5 Gy in 1 week is 31.3 Gy is similar to 10 × 3 Gy, one can expect similar LPFS. The EQD2 of 5 × 5 Gy for radiation-related myelopathy is 43.8 Gy (alpha/beta value of 2 Gy), which is below the tolerance dose of the spinal cord (45-50 Gy) [9–11].

In contrast to other countries, decompressive surgery prior to RT became increasingly popular for MSCC in Germany during recent years, although it is recommended only for selected patients [12–15]. Thus, the proportion of patients treated with RT alone for MSCC in Germany is decreasing, and a randomized, prospective clinical trial comparing 5 × 5 Gy of high-precision RT to 5 × 4 Gy of conventional RT with a sufficient sample size will be difficult to perform within a reasonable period of time. Therefore, the present study is designed as a single-arm phase 2 study. Subsequently, the patients of the phase 2 study will be compared to a historical control group. Propensity-score matching will be performed to balance covariates and remove bias that may arise due to these confounders [16]. Ten important potential prognostic factors will be included in the propensity-score [17]. This design can be considered appropriate to answer the question whether high-precision RT with 5 × 5 Gy results in significantly better LPFS than 5 × 4 Gy of conventional RT in patients irradiated for MSCC.

If superiority regarding LPFS can be shown for high-precision RT with 5 × 5 Gy, patients with MSCC would benefit from this regimen, since they can achieve high LPFS rates with an RT regimen lasting only 1 week (5 × 5 Gy) instead of 2 weeks (10 × 3 Gy). This study aims to make a significant contribution to the most appropriate RT schedule for patients with MSCC.

## Methods/Design

### Endpoints of the study

The primary endpoint is LPFS of MSCC after 5 × 5 Gy of high-precision RT. LPFS is defined as freedom from both progression of motor deficits during RT and an in-field recurrence of MSCC following RT leading to new or progressive motor deficits. It is supposed that 5 × 5 Gy results in better 6-month LPFS than conventional RT with 5 × 4 Gy. The following endpoints will be evaluated directly after RT and at 1, 3 and 6 months following RT: Motor function/ability to walk, sensory function, sphincter dysfunction, LPFS, overall survival (OS), pain relief, quality of life, and toxicity.

### Study design

This is a single-arm study, which will investigate the effect of high-precision RT with 5 × 5 Gy on LPFS in patients irradiated for MSCC. The recruitment of all 44 patients (40 patients +10% for potential drop-outs) should be completed within 18 months. The follow-up period will be 6 months. Another 6 months are required for analyses, reporting and publication. This equals a total running time for the study of 30 months. In accordance with a previous study assessing local control of MSCC, the following patient characteristics will be recorded to allow adequate comparison with the historical, propensity-score matched control group [16, 17]: Age, gender, type of primary tumor, interval from tumor diagnosis to MSCC, number of involved vertebrae, other bone metastases at the time of RT, visceral metastases at the time of RT, time developing motor deficits prior to RT, ambulatory status prior to RT, and Eastern Cooperative Oncology Group (ECOG) performance score.

### Inclusion criteria

1. Motor deficits of the lower extremities resulting from MSCC (may affect single or multiple spinal sites), which have persisted for no longer than 30 days
2. Confirmation of diagnosis by magnetic resonance (MR)-imaging (computed tomography (CT) allowed)
3. Age 18 years or older
4. Written informed consent
5. Capacity of the patient to contract

### Exclusion criteria

1. Previous RT or surgery of the spinal areas affected MSCC
2. Symptomatic brain tumor or symptomatic brain metastases
3. Metastases of the cervical spine only
4. Other severe neurological disorders
5. Pregnancy, Lactation
6. Clear indication for decompressive surgery of affected spinal areas

### Treatment

Radiotherapy is administered as high-precision radiotherapy with 25.0 Gy in 1 week, i.e. with 5.0 Gy per fraction on 5 days per week (representing an EQD2 of 43.8 Gy for radiation myelopathy) [6, 7]. An EQD2 of 45 Gy is estimated to be associated with a risk of radiation-related myelopathy of 0.03% and is therefore considered safe [8]. VMAT (6-10 MeV photon beams) is the preferred technique. SBRT is allowed for patients with involvement of only one vertebra, if the following constraints can be met. The clinical target volume (CTV) includes the vertebral and soft tissue tumor as seen on the planning computed tomography and diagnostic MR-imaging, the spinal canal, the width of the involved vertebrae, and half a vertebra above and below those vertebrae involved by MSCC. The planning target volume (PTV) should include the CTV plus 0.8 cm and should be covered by the 95%-isodose. The maximum relative dose allowed to the spinal cord is 101.5% of the prescribed dose (representing an EQD2 of 44.9 Gy for radiation myelopathy). This maximum dose is estimated to be associated with a risk of radiation-related of <0.03% and is, therefore, also considered safe [8]. Both the EQD2 of the prescribed dose (41.7 Gy) and the EQD2 of the maximum dose (43.8 Gy) are well below the tolerance dose of bone [9–11]. In accordance with the Quantitative Analyses of Normal Tissue Effects in the Clinic (QUANTEC) data, the mean EQD2 for esophagus, heart and lung must be <34 Gy, <26 Gy and ≤7 Gy, respectively [9]. Taking into account a radiation regimen of five fractions, the corresponding mean doses per fraction are 4.5 Gy, 3.8 Gy and 1.54 Gy, respectively [6, 7]. MSCC may affect single or multiple spinal sites. All sites are treated with high-precision RT. It is recommended that the patients receive concomitant dexamethasone during the period of radiotherapy if indicated [1, 2].

### Assessments

The following parameters will be recorded at the start of the study (baseline): Date of birth, gender, time between onset of motor deficits and start of RT, type of imaging used for diagnosis of MSCC, interval between initial tumor diagnosis and MSCC, dexamethasone treatment, surgical consultation, localization and number of involved vertebrae, type of primary tumor / histology, presence of other bone metastases or visceral metastases, performance status, motor function / ambulatory status (according to the modified Tomita scale [18]), sensory function, sphincter dysfunction, pain score, and quality of life (QoL) score [19]**.**

#### Local progression-free survival (LPFS)

LPFS time will be calculated from the last day of the radiotherapy treatment and assessed clinically directly after RT and at 1, 3 and 6 months following RT. In case of a suspected recurrence of MSCC (i.e. progression of existing or development of new motor deficits), a spinal MR-imaging will be performed to confirm or exclude an in-field recurrence of MSCC at any time. The number of MR-imaging sessions is minimized to clinically relevant situations, since patients with MSCC are often quite debilitated. Thus, diagnostic procedures, which may be burdensome for the patients, may not be performed for study purposes alone.

#### Motor function / ability to walk

Motor function will be evaluated using the following scale according to Tomita et al. [18] prior to RT, at the end of RT, and at 1, 3 and 6 months following RT: 0 = normal strength, 1 = ambulatory without aid, 2 = ambulatory with aid, 3 = not ambulatory, 4 = complete paraplegia. Improvement or deterioration of motor function was defined as a change of at least 1 point.

Motor function will additionally be evaluated separately for each leg using the following scale in reference to the American Spinal Injury Association (ASIA) classification [20] resulting in total points of 0 to 14: 0 = complete paraplegia, 1 = palpable or visible muscle contractions, 2 = active movement, without gravity, 3 = active movement, against gravity, 4 = active movement, against mild resistance, 5 = active movement, against intermediate resistance, 6 = active movement, against strong resistance, 7 = normal strength. Improvement or deterioration of motor function was defined as a change of at least two points.

#### Sensory function / sphincter dysfunction

Sensory function will be evaluated using the following scale, modified in accordance to the ASIA classification [20]: 0 = absent, 1 = impaired, 2 = normal, 9 = cannot be assessed. Sphincter dysfunction will be evaluated as yes vs. no.

#### Overall survival (OS)

OS time will be calculated for each patient from the last day of radiotherapy up to 6 months following RT. Patients will be followed up until death or for at least 6 months.

#### Pain relief

Vertebral pain will be evaluated with a numeric rating scale (self-assessment by patients) from 0 to 10 points (0 = no pain, 10 = worst pain) prior to RT and directly, 1, 3 and 6 months following RT. Improvement by two points is rated partial response, 0 points complete response. Pain will be assessed prior to RT and directly and 1, 3 and 6 months following RT.

#### Quality of life (QoL)

QoL will be assessed using the distress thermometer [19]. Patients can rate their impairment in QoL between 0 and 10 (0 = no, 10 = maximum impairment). QoL will be assessed prior to RT and directly and 1, 3 and 6 months following RT. Improvement in QoL was defined as improvement of at least two points compared to the QoL prior to RT (baseline).

#### Toxicity

Toxicity will be assessed Common Terminology Criteria for Adverse Events (CTCAE) version 4.3 during RT, directly after RT and at 1, 3 and 6 months following RT [21].

### Comparisons to historical control group

The patients of this study who received high-precision RT with 5 × 5 Gy for MSCC will be matched (propensity-score matching) to a historical control group consisting of about 400 patients treated with 5 × 4 Gy of conventional RT between 2001 and 2016. The patients of the control group are part of an already existing anonymized database. To be eligible for control group, patients fulfilling the same inclusion criteria and exclusion criteria as defined in the prospective phase 2 study are considered. Furthermore, to be consistent with efficacy analysis of phase 2 study, patients of the historical control group must have received at least 80% of the planned RT dose. This will lead to roughly 400 patients qualifying for the comparison with the prospectively collected phase 2 data. For comparison purposes, a propensity-score approach will be applied to account for baseline differences between treatment arms to balance covariates and remove bias that may arise due to these confounders. Covariates to be included in the model will be the following ten prognostic factors [16, 17]: Age (2 groups, depending on median age), gender, type of primary tumor (breast cancer vs. prostate cancer vs. myeloma/lymphoma vs. lung cancer vs. other tumors), interval from tumor diagnosis to MSCC (≤15 months vs. >15 months), number of involved vertebrae (1-2 vs. ≥3), other bone metastases at the time of RT (no vs. yes), visceral metastases at the time of RT (no vs. yes), time developing motor deficits prior to RT (1-7 days vs. 8-14 days vs. >14 days), ambulatory status prior to RT (no vs. yes), and Eastern Cooperative Oncology Group (ECOG) performance score (1-2 vs. 3-4).

### Sample size calculation

The primary goal of this study is to assess high-precision RT with 5 × 5 Gy in 1 week with respect to 6-month LPFS and to demonstrate that this rate is superior to conventional RT with 5 × 4 Gy. With respect to tumor cell kill, the EQD2 of 5 × 5 Gy is similar to the EQD2 of 10 × 3 Gy (31.3 Gy vs. 32.5 Gy) and higher than the EQD2 of 5 × 4 Gy (23.3 Gy). In a previous prospective non-randomized study, the 6-month LPFS rates were 86% after longer-course RT and 67% after short-course RT, respectively (*p* = 0.034). In that study, 95 of 117 patients (81%) in the longer-course RT group had received 10 × 3 Gy, and 91 of 114 patients (80%) in the short-course RT group 5 × 4 Gy. Assuming for the present study that conventional RT with 5 × 4 Gy in fact results in a 6-month LPFS rate of 67%, an increase by roughly 20 percentage points is considered clinically relevant and appears realistic when applying high-precision RT with 5 × 5 Gy.

The sample size is chosen to firstly obtain prospective phase 2 data that can be interpreted on its own and to secondly allow for comparison with historical data: A sample size of at least 40 eligible patients is needed to estimate the probability of LPFS at 6 month with adequate precision, based on the following assumptions: 6-month LPFS can be assumed to be 87%, 6-month LPFS estimated with a precision of +/− 20 percentage points expressed as the half length of the associated two-sided confidence interval with a confidence coefficient of 95%, and power of least 80%. Assuming that 10% of enrolled patients will not be eligible for efficacy analysis, 44 patients should be enrolled in the prospective part of this phase 2 trial.

The confirmatory study aim is to compare the prospectively collected phase 2 data with a historical, propensity-score matched cohort collected up to the time of data analysis [16]. Assuming for simplicity and conservative power calculation that this comparison could be conducted with a simple Pearson-Chi-Square test using a two-sided significance level of 5% (10%), a power of 79% (86%) is reached, if 40 patients are treated with high-precision RT and roughly 400 patients of the historical control group qualify for Propensity-Score adjusted comparison and assuming that the expected 6-month LPFS are 87% and 67%, respectively. Taking into account that the more sophisticated propensity-score adjusted statistical analysis will increase statistical power, the power for treatment arm comparison reached with 40 eligible patients in the prospective phase 2 part of the study can be assumed to be at least 80%.

## Discussion

For many patients with MSCC, who are suffering from severe pain and neurologic deficits, each radiation session may be associated with significant discomfort. Therefore, it appears reasonable to keep the number of treatment sessions and the overall treatment time as short as possible. Several radiation programs are available for the treatment of MSCC including single-fraction regimens and multi-fraction regimens including up to 20 fractions and lasting up to 4 weeks [2]. Single fraction programs can only be recommended for patients with a very poor survival prognosis of only a few weeks. Multi-fraction regimens are more appropriate for most patients with MSCC. If one aims to deliver a short-course program lasting only 1 week, e.g. 5 × 4 Gy, one has to be aware that the risk of an infield recurrence of MSCC in the irradiated spinal part is higher than with the most commonly used longer-course regimen 10 × 3 Gy [5, 22]. This advantage of 10 × 3 Gy over 5 × 4 Gy is most likely due to its higher EQD2 (32.5 Gy vs. 23.3 Gy) [6, 7]. A fractionation regimen that takes into account both aspects, i.e. a short overall treatment time of about 1 week and an EQD2 > 30 Gy, would be an ideal option for patients with MSCC. Such a regimen would be 5 × 5 Gy, which means that an EQD2 of 31.3 Gy will be delivered in only 1 week. However, 5 × 5 Gy can be absolutely safely administered only with the use of modern high-precision radiotherapy such as VMAT or SBRT. In order to be below the lower margin of the tolerance dose of the spinal cord, which is reported to be 45-50 Gy, the maximum dose to the spinal cord should not exceed 101.5% of the prescribed dose (5 × 5 Gy) [9–11]. This may be a challenge for the planning medical physicists and the planning process and, therefore, may take more time than for patients with MSCC receiving conventional RT. However, in the patients who have been included in the PRE-MODE trial so far, the complete process of treatment planning including computed tomography, contouring by radiation oncologist and planning by medical physicists did not hamper that the patients received their first radiation fraction within 24-48 h after their first presentation to a radiation oncologist, which is generally recommended time interval between first presentation and start of radiotherapy for patients with MSCC [2].

Important endpoints in the treatment of MSCC include among others the LPFS [2, 5, 17, 22]. An in-field recurrence of MSCC associated with neurologic deficits may cause a severe problem for the patients, since decompressive surgery with stabilization may not be possible or indicated, and a second course of radiotherapy may not be possible when considering the EQD2 of the first course of radiotherapy and the tolerance dose of the spinal cord [9–12]. Since the maximum dose delivered to the spinal cord for patients with MSCC is usually higher than 100%, which accounts for both total dose and dose per fraction, the EQD2 to the spinal cord is often significantly higher than the prescribed dose and may not allow a safe delivery of a second course of radiotherapy. Therefore, an in-field recurrence of MSCC must be avoided. Longer-course programs such as 10 × 3 Gy in 2 weeks have a higher EQD2 for tumor cell kill and result in better LPFS rates than short-course programs such as 5 × 4 Gy in 1 week [5, 22]. The fractionation regimen of the present PRE-MODE trial, 5 × 5 Gy, combines RT with a higher EQD2 (very similar to 10 × 3 Gy) and a short overall treatment time (same as 5 × 4 Gy). Therefore, this trial has the potential to make a significant contribution to the treatment of MSCC by sparing one week (50%) of the overall treatment time without impairing LPFS.

## Abbreviations

ASIA: American Spinal Injury Association
CT: Computed tomography
CTCAE: Common Terminology Criteria for Adverse Events
CTV: Clinical target volume
ECOG: Eastern Cooperative Oncology Group
EQD2: Equivalent dose in 2 Gy fractions
LPFS: Local progression-free survival
MR: Magnetic resonance
MSCC: Metastatic spinal cord compression
OS: Overall survival
PTV: Planning target volume
QoL: Quality of life
QUANTEC: Quantitative Analyses of Normal Tissue Effects in the Clinic
RT: Radiotherapy
SBRT: Stereotactic body radiotherapy
VMAT: Volumetric modulated arc therapy
